# First Processing Steps and the Quality of Wild and Farmed Fish

**DOI:** 10.1111/j.1750-3841.2010.01900.x

**Published:** 2011-01

**Authors:** Antonio J Borderías, Isabel Sánchez-Alonso

**Keywords:** bleeding, filleting, fish, gutting, primary processing, washing

## Abstract

First processing steps of fish are species-dependent and have common practices for wild and for farmed fish. Fish farming does, however, have certain advantages over traditional fisheries in that the processor can influence postmortem biochemistry and various quality parameters. This review summarizes information about the primary processing of fish based on the influence of catching, slaughtering, bleeding, gutting, washing, and filleting. Recommendations are given for the correct primary processing of fish.

## Primary Processing

### Wild fish

Wild fish are harvested by a large variety of methods, such as different kind of nets, hooks, pots, and so on. Depending on the method used the capture of wild fish involves various degrees of desperate struggle followed by a period of asphyxiation once the fish is on board. To control stress produced by these conditions it is necessary to control mainly the fishing method and time; however, the method is often dictated by commercial considerations, and it is difficult to modify. Incorrect handling at this point will be detrimental to the quality during ice storage. Fish that have been trawled are subject to more stress from fighting the net for hours, and this stress has been shown to affect ice-storage quality. In the case of some species like tuna, when they are caught in a highly stressed state, the build up of lactic acid in the muscle, combined with high muscle temperatures results in a dull muscle and acidic and metallic aftertaste (Goodrick 1987). This has been reported in other species; for instance, wild salmon caught by gill netting die after stress exhaustion. In these conditions *rigor mortis* is faster and quality result poorer (Dassow 1976). With harvesting by hook and line, when the fish is killed faster, there is less stress and the quality is better preserved during icing. In Atlantic cod (*Gadus morhua* L.), Botta and others (1987) reported that the fishing method is more significant than the season of capture.

From the point of view of contamination, Shewan (1949) reported that in general trawled fish carry microbial loads 10 to 100 times higher than line-caught fish because of mud stirring contamination and gut contamination produced by the pressure of the fish in the net. Moreover, in prolonged trawls the cod end becomes very full and the fish there can die of the pressure and become bruised. In this way, microorganisms can be introduced to the flesh, which is sterile just after death, thus accelerating spoilage (Costakes and others 1982).

It is important that fish be rapidly cooled and handled carefully as soon as it is on board. Liquid ice, made up of millions of ice “micro-crystals” formed and suspended within a solution of salt–water, is a good means of immediate rapid cooling because its temperature is lower than traditional ice and temperature transmission is faster in a liquid medium than in a solid one like normal ice (Reynisson and others 2010). Fish should not be exposed to the sun or the wind but should be carefully cleaned and cooled as soon as possible (FAO 1973).

### Farmed fish

Handling of farmed fish has certain differences compared to wild fish and depending of species. The first operation for farmed fish is to carefully separate fish from the main cages into smaller holding units without causing more stress than necessary. At this stage, the fish are kept at a density of around 5 to 10 kg/m^3^ until ready for collecting them (cropping). In live tilapia (*Oreochomis* sp.), ozone pretreatment with 6 ppm ozone in water during one hour prolongs shelf life by 12 d and improves the quality characteristics during storage at 0 °C for 30 d but has little effect at 5 °C (Gelman and others 2005; Glatman and others 2006). The improvement they report is probably the result of an initial reduction of spoilage bacteria and subsequent prevention of growth.

The second very important operation is starvation for as long as is necessary to ensure that gut contents are evacuated. During feeding periods, the digestive tract of the fish contains many bacteria that produce digestive enzymes capable of causing intense postmortem autolysis, resulting in strong odors and flavors, especially in the abdominal area (Huss 1995). By reducing the amount of feces in the intestines, spoilage is delayed, and digestive enzyme activity is reduced. If further processing steps are considered, for example, filleting and freezing, feeding interruption may be a determinant of the product shelf life (Huidobro and Tejada 2004). Starvation is also very important to prevent feces trailing from the anus, which is off-putting for consumers. In general, the starvation period is 1 to 3 d depending on temperature. Ferreira Pinto and others (2007) observed that 1 d was considered to be the minimum feeding interruption period in sea bream, with 8 d being the maximum. Reasons for extending this period in some cases beyond 48 h included variations in the market price for the fish and the time needed to empty the fishpond. One problem is that around 1% of weight can be lost due to starvation at higher temperature than 20 °C. Mechanical properties of sea bream muscle change as the starvation time progresses, so the flesh is firmer when they are starved for up to 8 d compared to the standard 1 to 3 d, due to changes in protein solubility and pH (Gines and others 2002).

## Stress Influence on Quality

Stress in wild and farmed fish, which are very active before slaughter, can affect the quality of the fish in a physical and a biochemical way (Robb 2001). From a biochemical point of view, if the fish is killed after muscle activity, its cells will contain more lactic acid from anaerobic respiration, so that adenosine triphosphate (ATP) synthesis is stopped and *rigor mortis* sets in sooner (Korhonen and others 1990; Lowe and others 1993). Spiking to instantaneously destroy the brain by puncturing, and so prevent muscle activity, delays the onset of *rigor mortis* as compared to a slower death such as immersion in chilled water. This happens because it retards the drop in ATP, which is the agent that prevents interlocking of thin and thick filaments (Boyd and others 1984). However, there is no difference in the final postslaughter pH of stressed and unstressed fish of the same species, despite differences immediately postmortem (Robb 1998).

The degree of muscle activity prior to slaughter also affects how firm the flesh becomes during *rigor mortis.*Mishima and others (2005) report that lactic acid concentration is lowest when horse mackerel (*Trachurus japonicus*) are killed by spinal cord destruction as opposed to other slaughtering methods, such as struggling and temperature shock; this slow *rigor mortis* onset results in slower muscle degradation in the course of iced storage, as measured by the ratio between nucleotides and nucleosides, degradation products from ATP, which is called the K-value. Stien and others (2005) report that a high storage temperature masks most of the effects produced by preslaughter stress; however, it is important to follow the stress management protocols when fillets are kept at the common storage temperatures under 4 °C. The temperature of the fish just after death will affect the course of various biochemical reactions during storage. This is caused by the reduction in ATPase activity as the temperature decreases, and by a reduction in the uptake of Ca^++^ (Robb 2001). Also, there are species-related differences; for instance Watabe and others (1989) found that onset of *rigor mortis* was temperature-dependent in plaice (*Paralichthys oliaceus*), whereas in horse mackerel Mochizuki and Sato (1994) found almost no differences between 0 and 8 °C.

In highly stressed fish, all muscles enter rigor very quickly and at the same time. As a result, the whole fish is very stiff and difficult to process. In fish with a low level of activity, only some muscles have been used and these are the ones that first enter *rigor mortis*, while the others do so later. Because of this difference in timing, not all the muscles are in *rigor* at the same time, so that the fish as a whole is less stiff (Robb 2001).

*Rigor mortis* has other consequences for muscle quality. For instance, fish which have been stressed before death present a considerable amount of gaping that is when myotomes separate from one another (Suzuki 1981). This is because the intervening threads of connective tissue break causing slits or holes to appear in the fillet. In severe cases, the fillet may even fall apart when skinned. This makes it more difficult to process the flesh, especially in the case of smoked salmon, where thin slices are required. Rough handling of fish can cause damage, which may result in gaping (Love 1974). The processing temperature is also important with regard to gaping. The connective tissue of newly caught fish is very sensitive to small rises in temperature, so when fish are warm, any handling such as gutting, washing, or moving can result in gaping. However, when warm fish are cooled again in ice, the connective tissue recovers most of its strength, unless the temperature has risen to about 30 °C, in which case the connective damage is irreversible (Love 1974). Some types of species are more susceptible to gaping than others; for example round fish like cod (*Gadhus morhua*), haddock (*Melanogrammus aeglefinus*), and salmon (*Salmo salar*) generally gape more than flat fish, and some species, for example, catfish never gape. Size also influences susceptibility to gaping; smaller fish seem to gape more because the connective tissue is thicker in larger fish (Love 1974). The season of capture is also important as regards gaping; for instance, when fish begin to feed heavily again after spawning, there is a general alteration of their biochemistry so that the myocommata are weakened, and the fish are very liable to gape (Morkore and Rorvik 2001).

Premortem handling stress significantly affects several color parameters of salmonids flesh (Robb and Frost 1999; Erikson and Misimi 2008); this loss of color is caused by insolubilization of muscle proteins as a result of low pH and subsequent drip loss occurred in the prerigor and development of *rigor mortis.*

## Stunning and Slaughtering Methods

Wild fish after capture follow a period of asphyxiation on board until they die. In farmed fish, the method most farmers use is to plunge the fish directly into iced water. It is very important to check that the water is kept close to 0 °C at all depths. If the temperature should rise to 8 °C, the fish will not die of thermal shock but of asphyxia, and this will adversely affect as their appearance, color, and texture. In the case of sea bass, it is very important that crowding prior to cropping be kept to a minimum and that fish are swiftly killed (Smart 2001), otherwise considerable damage, such as de-scaling and other skin lesions, and bleeding around the belly can occur.

Slaughtering by electrical stunning can produce enough active movements to break vertebrae, rupture blood vessels (Kestin and others 1995), which can result in blood spots (Van de Vis and others 2003). On the other hand Robb and others (2003) recommend electricity as better method for salmon stunning than carbon dioxide as this causes an earlier onset and resolution of *rigor mortis*. Sigholt and others (1997) reported that a sensory panel test differentiated between stressed and unstressed salmon killed by carbon dioxide stunning. They found that the texture of the stressed fish was softer during storage, which is detrimental especially when slicing smoked salmon. Kiessling and others (2004) also compared slaughtering of salmons with CO_2_ and with iso-eugenol. They found that although there are no differences in gaping, the meat is much softer when CO_2_ is used. Iso-eugenol (AQUI-S™ New Zealand, LTD) continues to be used for preslaughter sedation of salmon in the aquaculture industry in New Zealand, in spite of concerns by Japanese consumers about flavor residues (Gregory 2005). Roth and others (2007) report that percussive stunning, biting on the head, is the optimal choice for meat quality of turbot (*Scophthalamus maximus*), but electric stunning by prolonged electric exposure is also good. On the other hand, Poli and others (2005) report that asphyxiated and electrically stunned fish were more stressed than spiked, knocked, or live-chilled fish.

Van de Vis and others (2003) reported that eel slaughtering by a quick method like electricity in combination with nitrogen gas results in a better quality of meat than for instance the so-called salt bath, in which eels take about 20 min to die. On the other hand, Tejada and Huidobro (2002) reported that 3 slaughter methods were tested on farmed gilthead seabream (*Sparus aurata*): immersion in an ice salt–water slurry, asphyxia in air, and percussive stunning followed by immersion in ice and water. The result of this study was inconclusive as the use of different methods had no clear influence on the meat quality.

## Bleeding

Bleeding is recommended by several sources (Storm and Lien 1984; Valdimarsson and others 1984). On the other hand, other researchers (Meyer and others 1986; Moser 1986) have concluded that bleeding before gutting and gilling have no effect on parameters such as sensory, color, trimethylamine, and hypoxanthine concentration and surface bacterial load test. In the bibliography, there are many discrepancies about bleeding, this is the reason why it is not universally applicable, and that there are many factors to be considered, such as the type of species, the size, the season of capture, and so on.

Although bleeding is not generally recommended in wild fish, the bleeding produced should be profuse and should be done immediately after the fish is caught. Thus, some researchers (Botta and others 1986) have shown that bleeding is only effective in Atlantic salmon if conducted within 1 to 2 h of capture. Sohn and others (2007) report that simply removing a portion of the blood from live yellowtail by bleeding, is not sufficient to prevent lipid oxidation in the early stages of ice storage.

Bleeding is frequently used in farmed fish. Robb (2001) reported that large farmed fish needed to have the blood removed from the muscle and recommended cutting the gills with a sharp knife; this allows the fish to swim and so die from anoxia caused by blood loss. Robb and others (2003) concluded that exsanguinations generally reduce blood spots, but they could not say which methodology was better. They also reported that although bleeding affects the number of spots in smoked salmon, other factors can play an important role. In farmed halibut (*Hippoglossus hipoglossus*), Aske and Midling (2001) reported only small differences in hem iron muscle residues between bled and unbled lots, and they added that halibut killed by a blow to the head bled better than specimens anaesthetized with CO_2_.

Olsen and others (2006) reported that the amount of residual blood on salmon was influenced by the anaesthetizing and slaughtering procedures. Fish that were chilled alive and anaesthetized and then directly gutted had less residual blood in the fillet as compared to the standard industrial procedure of gill cutting and bleeding before gutting. Use of anesthesia on live-chilled fish killed by gill cutting did not result in a reduction of residual blood as compared to live-chilled unanesthetized fish killed by gill cutting. This was because blood coagulation time was prolonged at low temperature, possibly improving bleeding. Roth and others (2005) reported that the bleeding method was of less importance in trout and salmon than the timing of the bleeding. They observed no significant difference in blood spotting between fish that were bled live by a gill cut and those that were percussively killed and bled by gutting, so the industry would be well to gut directly. They also reported that drainage of blood in the fish muscle seemed to occur within the first hour postmortem, so that *rigor mortis* did not mediate in this processing.

## Gutting and Washing

The main reason for gutting is to prevent autolytic spoilage rather than bacterial spoilage (Shewan 1961). Industrial gutting and beheading are mechanized in developed countries today, but on board this operation is traditionally done by hand with a knife, and only in large ships are machines used. Gutting is usually done by cutting, but there are machines that perform gutting by sucking the viscera out and cleaning the belly part through the mouth. This method obviates the need to open the belly, but it makes it difficult to be sure how well the fish is cleaned. When gutting is performed, fish should be thoroughly washed to remove traces of blood and debris and to wash bacteria and intestinal content out of the gut cavity, skin, and gills of the fish. Erkan (2007) reported advantages when washing is included in the processing of sea bream. Other researchers, for example Samuels and others (1984), have reported that the practice of washing after gutting is more effective in removing remnants that in eliminating bacterial contamination. Kosak and Toledo (1981) reported that microbial decontamination with chlorine on the storage stability of iced finfish was highly effective. On the other hand Samuels and others (1984) found no advantage in dipping fish in a hypochlorite solution.

### Wild fish

Fatty fish like herring, sardines, sprat, mackerel, and so on are not gutted at sea because their small size and the large numbers in which they are caught make this impractical in the time available on typically short voyages to grounds not far from the port of landing. The washing equipment on small boats may consist simply of a hose and an open mesh basket, but large trawlers usually have a more sophisticated washing tank with circulating water. In these washers, the fish are discharged over a weir and down a chute to the fish room below deck. It is not clear that using large tanks is an advantage, for although they are effective in removing blood and debris, they can introduce a source of bacterial contamination. Scott and others (1986) compared microbiological and sensory assessment of whole compared to headed and gutted Orange Roughy (*Hoplostethus atlanticus*) during iced storage after washing with seawater. They found no significant differences in bacterial count between the 2 lots, but they did note a slight lengthening of shelf life in the gutted fish due to reduced autolysis. Working with Atlantic croaker (*Micropogon undulatus*) and grey trout (*Cynoscion regalis*), Townley and Lanier (1981) reported microbiological advantages in evisceration just after landing. A study by Meyer and others (1986) reported that the efficiency of pressure washing with surfactants in reducing surface flora in Atlantic mackerel (*Scomber scombrus*).

Another possible reason for gutting is to eliminate the possibility of parasite migration from the intestines into the muscle. In some countries, evisceration of different species has been established to minimize invasion of muscle (Chory 1988). On the other hand, Roepstorf and others (1993) showed that *Anisakis* larvae are already present in the flesh of herring at the time of capture. Immediate gutting on board cannot therefore eliminate or even reduce muscle infestation. Furthermore, Karl and others (2002) studied the possibility of migration of nematodes from the intestines into the muscle of ungutted pollack (*Pollachius pollachius*), haddock (*Melanogrammus aeglefinus*), and redfish (*Sebastes marinus*); their results showed that *Anisakis* larvae were already present in the flesh of all 3 species at capture, but no postmortem migration into the flesh was observed during 6 d of ice storage. Herreras and others (2000) also found *Anisakis* in muscle of hake (*Merluccius hubbsi*) that had been gutted just after the capture, indicating that worms had migrated into the muscle before capture. They also found that that density of *Anisakis* was significantly higher in the hypaxial muscles than in the epiaxial muscles, which means that the removal of hypaxial muscle can reduce the risk of A*nisakis* intake.

### Farmed fish

There is not a common opinion on the impact of the gutting practice on farmed fish. Cakli and others (2006) found psycrophilic counts in sea bream (*Sparus aurata*) and sea bass (*Dicentrarchus labrax*) a little lower in ungutted than in gutted fish; nevertheless, the quality as gauged by taking chemical, sensory, and microbial tests was similar throughout iced storage. Erkan (2007) reported that shelf life was similar for gutted and whole sea bream (*Sparus aurata*) on the basis of overall acceptability scores in sensory evaluation. Tejada and Huidobro (2002) reached a similar conclusion in the same species, reporting that gutting reduced the intensity of *rigor mortis* and the microbial load, although none of the other quality parameters were affected. On the other hand the results reported by Papadopoulos and others (2003), based on sensory and microbiological analyses, suggest that gutted sea bass have a shorter shelf life than ungutted specimens. Another reason for gutting is that gutted fish chill more rapidly as it happens in the case of albacore (*Thunnus alalunga)* (Price and others 1991). On the other hand, during gutting the belly area is exposed to air, which makes it more susceptible to oxidation (Røra and others 2001). Fed fishes spoil more quickly than starved ones when ungutted (Meyer and others 1986), because in the former proteolytic activity in the viscera will cause autolysis after death, possibly producing off-flavors or causing rib separation from the muscle or belly-burst (belly part broken).

## Packaging

The last operation on board is packaging, mostly in polystyrene or plastic boxes (utilization of wooden boxes is not common in developed countries), where fish is mixed with enough ice. Careche and others (2002) report that anchovy (*Engraulis encrasicholus*), a rather delicate fish, transported in water and ice in small polystyrene containers for 20 h, presented less spoilage than when stored in ice. This practice is also used for other small species like sardine (*Sardina pilchardus*) and blue whiting (*Micromesistius poutassou*) in southern Europe.

In farmed industry, fish must not warm up from the slaughter tank to the polystyrene or plastic boxes, which are filled with sufficient ice to maintain the temperature. Generally, fish are placed in boxes with the belly cavity upwards and fitted in to avoid unnecessary movement during transportation, which can be up to 10 to 15 d.

## Filleting

Many species are filleted to satisfy consumer demand. In general, filleting adds value to the product, although this depends very much on the type of market. Most companies that commercialize fillets use filleting-machines. Basically these machines cut along the upper and lower appendices on the spine, cutting the ribs, and vertebrae with a pair of symmetrical knives. Different standards of trimming are used, from removing only the backbone to removing visible fat, pin bones, and skin, and these different produce different yields. Fillet yield depends on the species, sex, size, and its structural anatomy (Røra and others 2001). Fish with large heads and frames relative to their musculature give a lower yield than those with smaller heads and frames. In farmed fish, yields can also be affected by farming conditions (feeding, water temperature, type of pond, and so on). Of commercially farmed fish species, tilapia (*Oreochromis* sp.) has the lowest fillet yield (33%) as compared to salmon (*Salmo salar*) (>50%), channel catfish (*Ictalurus punctatis*) (>38%), and striped bass (*Morone saxatilis*) (>40%). Sea bream and sea bass also give higher fillet yields than tilapia. Freshwater eel gives the highest fillet yield (60%).

Filleting is traditionally performed after the onset of *rigor mortis*, but this should be weighed against loss of freshness and the cost of storage. If fish is processed in *rigor*, the yield will be poor and it may cause gaping (Laverty 1984; Huss 1995). Large farmed species like salmon are usually filleted once *rigor* has been resolved, normally 3 to 4 d after death. On the other hand, Shaw and others (1984) reported that filleting after 7 d rendered the longest shelf life. In any case, it is difficult to industrially control the onset of *rigor* in large catches of wild species; fish farming makes it easier to control all the parameters that culminate in *rigor*.

Prerigor filleting has several advantages, one being that it ensures very fresh processed fish with little or no fillet gaping (Andersen and others 1994), although it changes the shape (Skjervold and others 2001; Kristoffersen and others 2007) and prerigor fillets are significantly thicker (Skjervold and others 2001). Also, the texture is softer than in rigor and certain operations such as the removal of pin bones are more difficult. The texture of prerigor fillets of farmed Atlantic cod (*Gadus morhua*) depends in part on dietary content; for instance, dietary inclusion of soybean oil has been found to result in faster reduction of breaking strength and darker muscle (Morkore 2006). However, there may be extensive loss of weight and proteins during subsequent storage if fish are filleted prerigor (Kristoffersen and others 2007). Prerigor processing then presents problems, but there are obvious advantages to processing immediately after slaughter: the products can be shipped to market 3 to 5 d earlier and a prolonged shelf life is a major economic benefit (Rosnes and others 2003; Tobiassen and others 2006).

## Recommendations

In wild fish, it is important to use methods of capture that do not exhaust the fish as is the case of harvesting by hook and line. Then, cooling with ice or chilled water should be performed as soon as possible and the fish handled with care. In farmed fish, quick slaughtering after nonstressful handling make for a more humane death and the product will have better quality and a longer storage life. Also, starvation is an important step in farmed fish.

An optimal slaughtering method from the standpoint of quality and welfare should render fish unconscious as soon as possible. Electrical stunning is an option, but blood spots sometimes appear on the muscle, so the electrical parameters need to be optimized. Prototypes for percussive stunning have recently been developed. In some species like gilthead sea-bream, there are no apparent differences in meat quality whether a quick or a slower slaughter method is used.

Bleeding is only practically applicable to medium-large species. The effect of bleeding is also controversial although the majority consensus, with which the authors agree, is that fish should be bled as soon as possible for a minimum of 30 min. The technique of having the fish swim with the gill cut seems a good method of bleeding, but it is not the best slaughtering method in terms of preventing the degradation of muscle ATP and retarding the onset of *rigor mortis*.

Gutting, like bleeding, is also applicable to medium and large species. There is doubt as to whether gutting is effective in removing microorganisms and parasites. What is very important is to wash thoroughly with water after gutting and to completely remove blood spots and debris in the gut cavity.

Filleting is primarily a means of food presentation intended to facilitate culinary preparation. Temperatures must be lower than 17 °C to avoid gaping in fillets, and the specific temperature threshold is species-dependent. Prerigor filleting ensures a very fresh product without gaping, but this practice is not always possible and presents some problems such as fillet deformation.
